# Glutamate and α-ketoglutarate: key players in glioma metabolism

**DOI:** 10.1007/s00726-016-2342-9

**Published:** 2016-10-17

**Authors:** Andreas Maus, Godefridus J. Peters

**Affiliations:** 1 Department of Medical Oncology, VU University Medical Center, PO Box 7057, 1007 MB Amsterdam, The Netherlands; 2 University of Gottingen, Gottingen, Germany

**Keywords:** Glioma, Glutamate, Alpha-ketoglutarate, Isocitrate dehydrogenase, Branched chain amino acids

## Abstract

Glioblastoma multiforme (GBM), or grade IV astrocytoma, is the most common type of primary brain tumor. It has a devastating prognosis with a 2-year-overall survival rate of only 26 % after standard treatment, which includes surgery, radiation, and adjuvant chemotherapy with temozolomide. Also lower grade gliomas are difficult to treat, because they diffusely spread into the brain, where extensive removal of tissue is critical. Better understanding of the cancer’s biology is a key for the development of more effective therapy approaches. The discovery of isocitrate dehydrogenase (IDH) mutations in leukemia and glioma drew attention to specific metabolic aberrations in IDH-mutant gliomas. In the center of the metabolic alterations is α-ketoglutarate (αKG), an intermediate metabolite in the tricarboxylic acid (TCA) cycle, and the associated amino acid glutamate (Glu). This article highlights the role of these metabolites in glioma energy and lipid production and indicates possible weak spots of IDH-mutant and IDH-wt gliomas.

## Introduction

Glutamic acid is an amino acid that plays an important role in energy metabolism and as excitatory neurotransmitter in the central nervous system. In the context of molecular biology the name of its carboxylate anion glutamate (Glu) is used synonymously, because in the body glutamic acid appears in its dissociated form. A link between Glu and energy metabolism was already described in the 1920s (Thunberg 1920). In 1936—Malherbe showed that the reversible oxidation of Glu to α-ketoglutarate (αKG) could be found in brain tissue. He further hypothesized that in vivo the enzyme “glutamic acid deaminase”, today referred to as glutamate dehydrogenase (GDH), executes the synthesis of Glu more often than its degradation. One part of this article will elucidate under which circumstances Weil-Malherbe’s assumption is true.

**Fig. 1:**
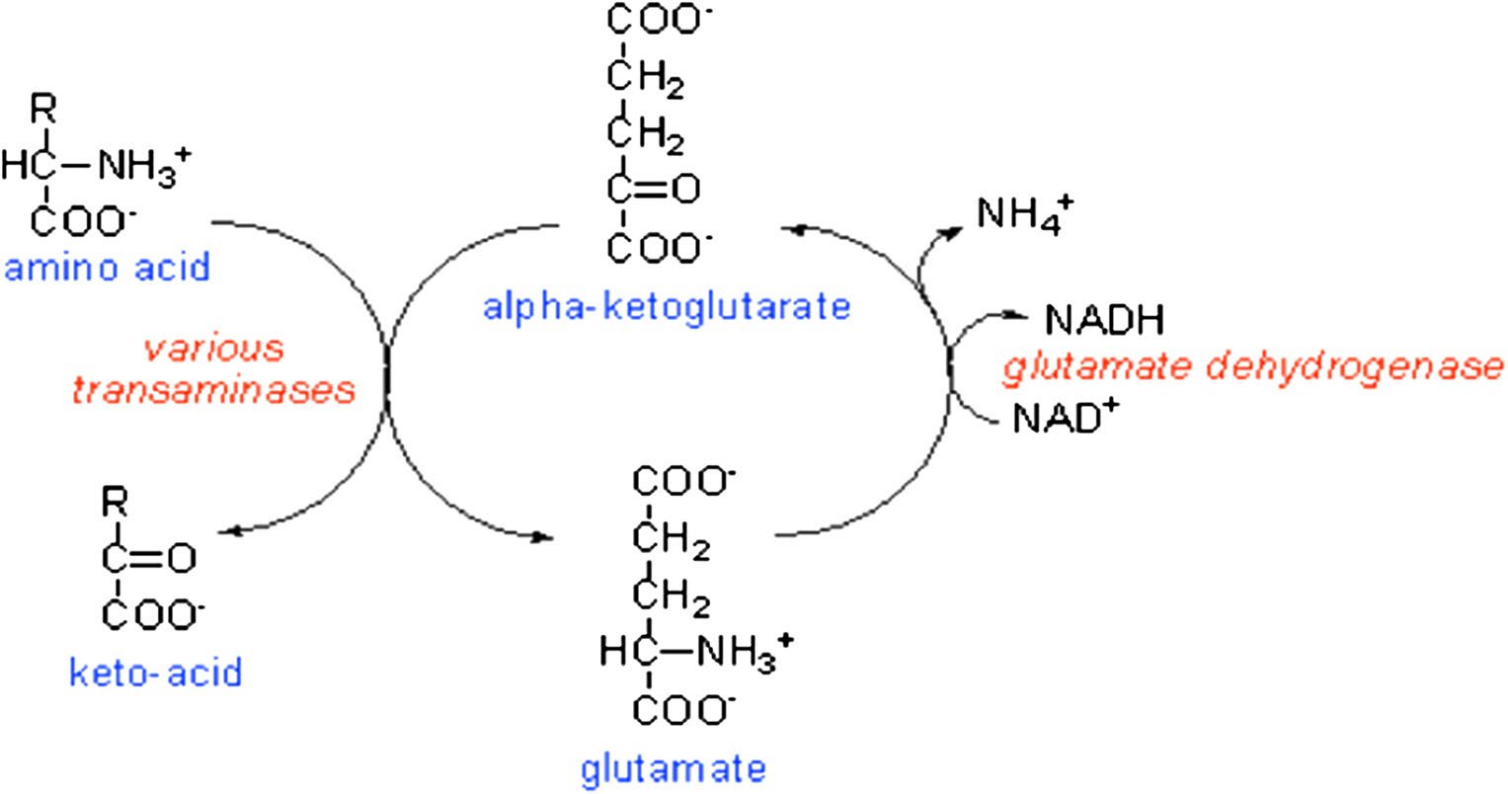
Glutamate dehydrogenase mediates the NADH-producing conversion of glutamate to α-ketoglutarate. From https://www.david-bender.co.uk (assessed 26-02-2016)

αKG is an intermediate metabolite in the tricarboxylic acid (TCA) cycle (or citric acid cycle, or Krebs cycle). The TCA cycle is an evolutionary conserved pathway. In eukaryotic cells it takes place in the mitochondria and serves mainly the production of ATP by breakdown of macromolecules. The reactions yield the reducing agents NADH and NADPH, which are used in the electron transport (oxidative phosphorylation) pathway for ATP production. In addition the TCA cycle is crucial for providing substrates for lipid and fatty acid synthesis. The transamination of αKG produces Glu and, dependent on the amino acid, a keto-acid (Fig. 1). Amino acids like alanine or aspartate are converted to pyruvate or oxaloacetate, respectively. These products are important metabolites in pathways like the TCA cycle or glycolysis. It became extremely relevant to the field of cancer metabolism when isocitrate dehydrogenase 1/2 (IDH1/2) mutations in glioma and acute myeloid leukemia were discovered (Dang et al. 2010; Reitman and Yan 2010). Mutated IDH enzymes have a shifted functionality, with which αKG becomes the main substrate for 2-hydroxyglutarate (2HG) synthesis.

Aside from oxidative deamination, Glu is often synthesized from glutamine (Gln). Due to its role as neurotransmitter, Glu is constantly released from neurons. Its synthesis and degradation are part of the glutamate/glutamine cycle. After release Glu is taken up by astrocytes, where it is converted to Gln and released again (Bak et al. 2006; Hertz 1979; Hertz et al. 1999). In the cytoplasm Gln can be converted to Glu by the enzyme glutaminase (GLA).

Gliomas account for 50 % of intracranial tumors and are the most common primary form of cancer in the brain. Glioblastoma multiforme (GBM; WHO grade IV astrocytoma) accounts for most of them. 12 % of GBMs carry a mutation in the *IDH1* gene, which encodes for the metabolic enzyme isocitrate dehydrogenase (Parsons et al. 2008). The discovery of *IDH* mutations in gliomas led the focus on cancer metabolism. A better understanding of tumor genesis could increase the hope of finding effective treatments against it. Better treatment strategies are urgently needed, because the prognosis for gliomas, especially for GBM is abject. Standard treatment for GBM includes resection via surgery, followed by radiation and adjuvant chemotherapy with the alkylating agent temozolomide. Although administration of temozolomide improves overall survival significantly, median survival ranges in between 12 and 15 months and the 2-year survival rate averages 26 % (Stupp et al. 2005).

## Glutamate and α-ketoglutarate in glioma metabolism

### The Warburg effect

Otto Warburg described a metabolic switch in cancer cells concerning the use of glucose (Warburg et al. 1926; Warburg 1925, 1954). In recent times the shift from pyruvate oxidation to lactic acid fermentation is known as the “Warburg-effect” and is considered a hallmark of cancer (Hanahan and Weinberg 2011). The metabolic change to lactic acid fermentation is baffling at first, because it leads to a smaller net ATP production than pyruvate oxidation and therefore seems like a reduction of the cell’s energy production. Today it is widely believed that the Warburg effect enables the cell to have building blocks like amino acids and nucleosides readily available. They are crucial for the synthesis of macromolecules and organelles, which enable the cancer cell to meet the special needs regarding fast growth and proliferation. The switch is accompanied by an increased glucose influx, possibly to make up for the less efficient ATP production.

### Glutaminolysis

Glu plays an important role as intermediate metabolite of glutaminolysis. Glutaminolysis describes the sequence of enzymatic reactions that turn glutamine (Gln) into substrates that enter the TCA cycle. The first step of this sequence is the hydrolysis of the amino group of Gln, which turns it into Glu. The reaction is mediated by glutaminase and can be displayed as: Glutamine + H_2_O → Glutamate + NH_3_. In cancer, glycolysis and glutaminolysis are the major mechanisms of ATP production, which means glucose and Gln are crucial nutrients. HIF1α activity can disrupt the function of the pyruvate dehydrogenase (PDH) complex (Kim et al. 2006). This will inhibit the introduction of glucose-derived metabolites into the TCA cycle and therefore renders the cell dependent on glutaminolysis. Glutaminolysis alone can rescue the cell from cell death. It is a key enzymic pathway for cancer metabolism, because it provides nitrogen for nucleotide and amino acid synthesis, it offers an alternative carbon source to supply TCA cycle intermediates, and as a byproduct NADPH is formed for lipid and nucleotide synthesis (reviewed by DeBerardinis and Cheng 2010).

### Reductive carboxylation

**Fig. 2:**
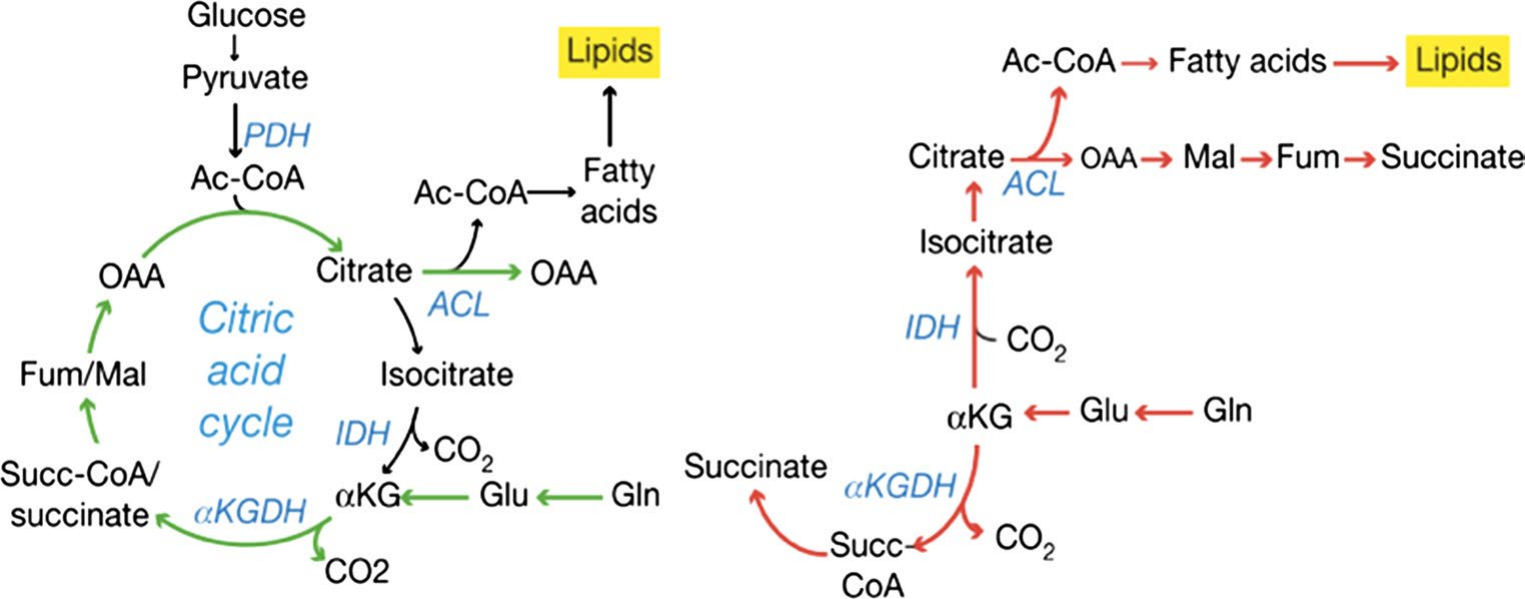
Intact TCA cycle (*green*). When PDH is blocked, or the TCA cycle is disrupted for other reasons, IDH-dependent reductive carboxylation sustains the formation of intermediate metabolites and fatty acids (*red*). From Mullen et al. (2012) with permission

Besides glutaminolysis, Gln can be a substrate for another distinct cytosolic pathway, which includes reversed flux through the TCA cycle. Where glutaminolysis mainly serves the production of energy in the form of ATP, reversed flux through the TCA cycle favors the synthesis of acetyl-CoA (Filipp et al. 2012). In regular cell metabolism glucose-derived pyruvate will enter the TCA cycle and then serves as the main source of acetyl-CoA, a precursor of fatty acids and lipids (Fig. 2). Under conditional aerobic glycolysis or under hypoxic conditions cells convert glucose to lactate. This will reduce the flux of pyruvate into the TCA cycle. Acetyl-CoA is then produced by breakdown of the TCA cycle-metabolite citrate. Hence, a lack of acetyl-CoA could also stem from disabled citrate formation by defective mitochondria, such as disruptions in the TCA cycle or electron transport chain (Mullen et al. 2012). In hypoxia HIF1α activity interferes with glucose carbon use in citrate synthesis by hampering PDH. A study performed with a GBM cell line in hypoxia (0.5 % O_2_) came to the conclusion that Gln is the major source for carbon under hypoxic conditions (Wise et al. 2011). Additionally, it was shown that reductive carboxylation of αKG (from Gln) is IDH2-dependent (Fig. 2). IDH2 is known to catalyze the oxidative decarboxylation of isocitrate to αKG. Studies indicate that it is also crucial for the reverse reaction (Wise et al. 2011; Mullen et al. 2012). Although the studies showed that IDH2 is crucial for sustained reductive carboxylation under hypoxia, they failed to assess the importance of IDH1 in that matter.

In contrast to these results it was shown that there is a TCA cycle-independent cytoplasmic pathway of reductive carboxylation of αKG, which is mediated by IDH1 (Metallo et al. 2012). This pathway, too results in the formation of isocitrate, citrate, and finally in acetyl-CoA for lipid synthesis.

Another positive effect of reductive carboxylation for the cancer cells is that it decreases ROS levels and produces NADPH as a byproduct.

### Glutaminase isoenzymes can exert contrary functions

As mentioned above, the first step of Gln degradation is catalyzed by glutaminase (GLA). The paralogous GLA isoenzymes are encoded by the *Gls* and the *Gls2* genes (reviewed by Campos-Sandoval et al. 2015). These genes are linked to tumor behavior, because oncogenes and tumor suppressor genes regulate them. Interestingly, differential expression of GLA isoenzymes alters the metabolism of nutrients. In brain tumor settings, however, the exact role of GLA isoenzymes is unclear, because these isoenzymes can exert contrary functions. For example, silencing the *Gls* gene (GLS) in glioblastoma cells LN229 and SFxL inhibited growth, lowered survival ratios, and induced apoptosis. The growth inhibition was even stronger under oxidative stress. Similar observations were made when the liver-type *Gls2* gene was *over*expressed in T98G glioma cells (reviewed by Campos-Sandoval et al. 2015).

### IDH wt glioma cells release Glu

**Fig. 3:**
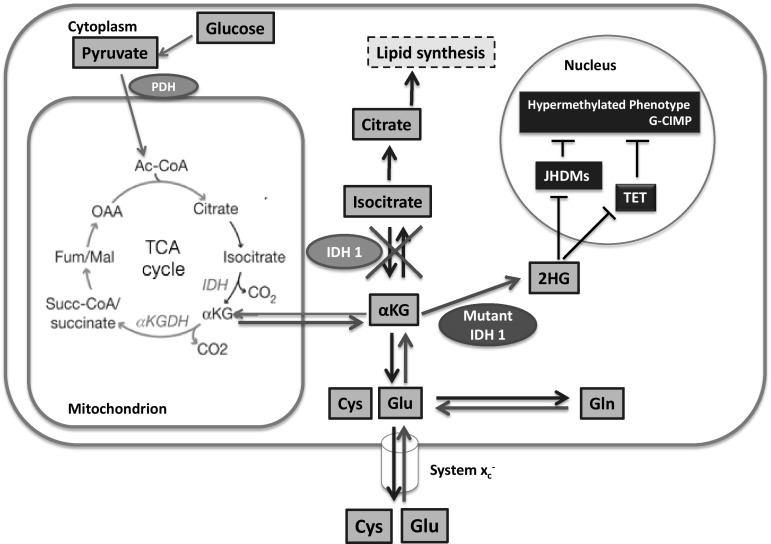
Overview of biochemical reactions for energy and lipid production in the cell. α-ketoglutarate (αKG) and glutamate (Glu) are in the center of pathways like glutaminolysis or reductive carboxylation. Mutant IDH1 changes the dynamics of metabolic processes in the cell and leads to accumulation of 2HG. 2HG in turn inhibits JHDMs and TET, which leads to a hypermethylated phenotype. Ac-CoA, acetyl-CoA; OAA, oxaloacetate; Gln, glutamine; Glu, glutamate; Cys, cysteine; αKG, α-ketoglutarate; Succ-CoA, succinyl-CoA; Fum, fumarate; Mal, malate; PDH, pyruvate dehydrogenase; ACL, ATP-citrate lyase; IDH, isocitrate dehydrogenase; αKGDH, α-ketoglutarate dehydrogenase; JHDMs, Jumonji domain-containing histone demethylases; TET, Ten-Eleven-Translocation

In IDH wild-type (wt) gliomas, Glu synthesis is catalyzed by high levels of branched-chain amino acid transaminase 1 (BCAT1), which convert αKG into Glu (Tönjes et al. 2013). The byproduct of this reaction is ammonia. When intracellular Glu levels rise, excessive Glu is released via the glutamine/cysteine antiporter System *x*_c_^−^ (extensively reviewed by Lewerenz et al. 2013) in exchange for cysteine (Cys; Fig. 3). This exchange is favorable for the cancer cells, because Cys is a major component of the antioxidant Glutathione (GSH), which in turn is an antagonist of reactive oxygen species (ROS). Elevated ROS levels trigger apoptosis; hence to antagonize ROS is important for cancer cell survival. There are external and internal causes for elevated ROS levels. The main external cause in GBM, radiotherapy, directly induces ROS through radiation. Internal causes of ROS lie in the altered metabolism itself. In cancer cells large amounts of glucose are oxidized in the TCA cycle, rendering the cell with superoxide anions as side products. These superoxide anions contribute to high ROS levels (Masui et al. 2014). High levels of GSH enable cancer cells to evade the induction of apoptosis through induced ROS.

### Methionine–Cysteine double deprivation increases ROS levels in glioma cells

Recently it was shown that deprivation of amino acids like methionine (Met) or Cys leads to induction of ROS, too. Met or Cys deprivation alone already resulted in decreased proliferation of cells of the glioma cell lines U87 and U251 (Liu et al. 2015). Double deprivation had a synergistic effect.

When extracellular nutrient supply is scarce, autophagy enables the tumor cells to meet the demand for required amino acids and nucleotides. Also the induction of ROS can induce autophagy in cancer cells (reviewed by Bellot et al. 2013). Liu et al. (2015) studied the effects of double deprivation on autophagy. Met–Cys double deprivation led to an increased abundance of the autophagy-related protein LC3-II and autophagosomes. Furthermore, inhibition of autophagy increased the sensitivity of glioma cells to double deprivation.

In vivo studies showed that glioma proliferation was lower in tumor-bearing mice that received a Met–Cys deprived diet compared to a standard diet. In addition, histologic examination of tumors from Met–Cys deprived mice showed signs of autophagy (Liu et al. 2015).

The induction of ROS in cancer cells through Met–Cys double deprivation offers a potential additional therapy option that would enhance the ROS inducing effects of chemo- and radiotherapy.

### High levels of extracellular Glu impair healthy cells in the tumor microenvironment

The exchange of Glu for Cys is favorable for the cancer cell, but not for healthy cells in its vicinity, because Glu causes cell death in the tumor environment (Takano et al. 2001; Ye and Sontheimer 1999). High levels of extracellular Glu lead to an imbalance in the glutamate/glutamine cycle between astrocytes and neurons. The results can be astrocyte swelling, a block of astrocytic Glu uptake and neuronal cell death (Bak et al. 2006; Jayakumar et al. 2006; Albrecht et al. 2006). The challenge for the astrocytes does not seem to be the origin of Glu, but the amount. Yao et al. (2014) showed that astrocytes were able to take up Glu that was released by glioma cells. In a co-culture of astrocytes and glioma cells with the ratio 1:1, Glu released by glioma cells was taken up by astrocytes and no neuronal damage was observed. In these cultures extracellular Glu levels were reduced to 1–5 μM (compared to +30 μM in single glioma culture). Glu uptake by astrocytes reduced glioma cell proliferation, and prevented neuronal death by Ca^2+^ overload. These conditions might resemble the tumor environment at an early stage of tumor development. In late stage gliomas, the astrocyte to glioma cell ratio is more likely to be around 0.5:1 or smaller. In vitro this ratio led to an increase of extracellular Glu and ammonia levels, which resulted in a block of Glu uptake by astrocytes and cell death (Yao et al. 2014).

### Block of system *x*_c_^−^ has positive effects on cells in the tumor vicinity

One approach to reduce the damaging effects of high extracellular Glu levels was to reduce the amount of secreted Glu by blocking the System *x*_c_^−^ (Savaskan et al. 2008). System *x*_c_^−^ is composed of xCT and CD98 in human primary gliomas and tested cell lines. In vitro Glu secretion was successfully reduced by downregulation of xCT via small interfering RNA (siRNA), and with the selective xCT inhibitor S-4-CPG (Savaskan et al. 2008). Reduction of xCT had no impact on morphology, cell cycle progression, ROS formation or proliferation Brain slices challenged with conditioned media from xCT-silenced cells showed significantly less cell death than the control with conditioned media from glioma cells. In vivo studies confirmed that inhibition of xCT through S-4-CPG reduced Glu secretion. That resulted in a lower neuronal damage, later onset of neurological deficits, and prolonged survival compared to the vehicle control group (Savaskan et al. 2008).

Intracellular peaks of acetyl-CoA levels correlate with phases of growth and proliferation in the yeast metabolic cycle. Furthermore, the gene battery in yeast that is differentially acetylated during high acetyl-CoA levels corresponds to target genes of c-Myc in mammalian cells (Ji et al. 2011). It has been concluded that acetyl-CoA levels directly influence epigenetic regulation through differential acetylation, which would describe an evolutionary conservative mechanism to link growth and proliferation to the nutritional state of the cell (Kaelin and McKnight 2013; Masui et al. 2014).

## Metabolic compensations to anti-tumor therapy

Key cancer metabolites have been the target of anti-tumor therapies, but most single target approaches have failed, because cancer cells can compensate for disturbed pathways and lacking metabolites. Here, we give some examples of adaptions made by glioma cells to deletion or impairment of metabolic pathways.

### Increased synthesis of asparagine halts apoptotic pathway in glutamine-depleted glioma cells

The non-essential amino acid Gln is, beside glucose, the most important energy source for glioma cells. Zhang et al. (2014) reported that Gln withdrawal led to apoptosis in SF188 human glioma cells with MYC amplification. That is somewhat surprising regarding the theoretical ability of the cell to synthesize Gln when sufficient amounts of glucose are available. Since Gln is crucial for asparagine (Asn) synthesis, it was tested if depletion of Asn has an effect on SF188 cells. Asn was depleted through knockdown of the enzyme asparagine synthetase (AS) that catalyzes the biosynthesis of Asn from aspartate and Gln. It has been reported that AS mRNA abundance is negatively correlated with glucose availability (Barbosa-Tessmann et al. 1999). AS depletion led to apoptosis in SF188 cells (Zhang et al. 2014). Addition of extracellular Asn completely restored survival and proliferation. These results are in line with the studies on the evasion of apoptosis through Asn in the case of sarcoma (Hettmer et al. 2015), and human melanoma and epidermoid carcinoma cells (Li et al. 2015). The authors conclude that Asn is crucial for cellular adaption to loss of Gln (Zhang et al. 2014). In Asn-deficient cells, translation of stress response RNAs like CHOP leads to apoptosis. Asn alone was sufficient to stop the apoptotic function of ATF4 through regulation of a pathway that induces translation-dependent apoptosis. Intracellular depletion of Asn alone resulted in apoptosis independent of Glu or glucose availability (Zhang et al. 2014).

Acute lymphoblastic leukemia (ALL) has been successfully treated through extracellular removal of Asn with l-asparaginase (Avramis 2012). Asparaginase (ASNase), an enzyme that hydrolyzes Asn, offers an option to deplete Asn intracellularly. Panosyan et al. (2014) showed that reduction of Asn through ASNase led to growth inhibition in DAOY medulloblastoma cells, GBM-ES cells, U87 cells, and mouse glioma (GL-261) cells in vitro. Gln addition increased Asn synthesis and abrogated the effect. Moreover, ASNase treatment affected the formation of neurospheres negatively.

Despite the promising in vitro results, ASNase-treated mice showed the same DAOY tumor growth as controls. Moreover, co-treatment with ASNase and temozolomide resulted in growth inhibition compared to temozolomide alone.

### Regulators of amino acid metabolism

Crucial amino acids can be acquired in various ways. The example of Asn shows that they can derive from breakdown of metabolites like Glu, or they can be taken up from the extracellular matrix. A more common source of free amino acids in the cytosol is the degradation and breakdown of proteins and peptides. This recycling process occurs constantly in healthy and transformed cells and is orchestrated by proteasomes and aminopeptidases (Saric et al. 2004). Aminopetidases such as serine aminopeptidase dipeptidyl peptidase (Busek et al. 2012), leucine aminopeptidase 3 (He et al. 2015) and methionine aminopeptidase 2 (Dasgupta et al. 2005) play a role in glioma. However, no treatment that involves aminopeptidases has been proven to be effective in glioma. However, there is no effective aminopeptidase inhibitor registered, while those currently in clinical development have not yet been tested in glioma (Hitzerd et al. 2014).

There are several approaches to inhibit the protein degradation pathway in cancer, such as E3 ubiquitin ligase inhibitors (Snoek et al. 2013), proteasome inhibitors, and aminopeptidase inhibitors (reviewed by Hitzerd et al. 2014). Some of these have been tested in clinical trials, or are clinically approved like the proteasome inhibitor bortezomib. Insights that were obtained in these trials could help to develop a specific strategy for glioma treatment.

### mTOR inhibitors have not been proven effective in glioma therapy

A central regulator of metabolism and cell growth is the kinase mechanistic target of rapamycin (or mammalian target of rapamycin; mTOR). mTOR is a downstream target of EGFR (epidermal growth factor receptor) through the PI3 K-Akt signaling pathway. 40 % of GBM have aberrant EGFR signaling; most carry the EGFRvIII mutant (Ekstrand et al. 1991; Wong et al. 1992). The mutant is characterized by a deletion of exons 2–7 of the *EGFR* gene, which results in an in-frame deletion of 267 amino acids from the extracellular domain of the receptor. EGFRvIII receptors are unable to bind growth factors, but are constitutively active in downstream signaling.

mTOR is found in two major complexes; mTOR complex 1 (mTORC1) and mTOR complex 2 (mTORC2). Both complexes promote increased c-Myc activity. mTORC1 splices the MYC-interacting protein MAX, which enhances c-Myc action (Babic et al. 2013). mTORC2 controls c-Myc levels in a FOXO-acetylation-dependent manner (Masui et al. 2013). Overexpressed Myc is a strong oncogene and has been explored in multiple types of cancer including glioma and GBM. It has been shown that c-Myc negatively regulates the tumor suppressor gene *PTEN* (Guo et al. 2013) and is involved in resistance to temozolomide therapy (Luo et al. 2015). Besides these effects it is strongly involved in metabolic reprogramming in glioma. It enhances GLS activity, which leads to increased glutaminolysis, higher Glu production and possibly increased reductive carboxylation.

Targeted therapies with mTOR kinase inhibitors have not proven to be effective in glioma therapy. To elucidate underlying mechanisms, magnetic resonance spectroscopy (MRS) of 12 GBM patients was carried out (Tanaka et al. 2015). The study indicated that Gln was strongly involved in metabolic reprogramming in GBM cells and the authors saw a connection between upregulation of Gln metabolism through increased GLS activity, and resistance to mTOR kinase inhibitors (Tanaka et al. 2015). The results indicated that mTOR-targeted therapy led to increased Gln metabolism and rendered glioma cells Gln-dependent, which could be an approach for effective therapy options. Combinations of mTOR inhibitors with other drugs affecting signaling pathways may have promise for further development.

Another way to inhibit mTOR signaling is the use of aminopeptidase inhibitors. Compounds such as tosedostat or bestatin inhibit aminopeptidase, which results in lowering the free amino acid levels. This in turn decreases mTOR signaling (reviewed by Hitzerd et al. 2014).

## Differences between IDH-wt and IDH-mutant gliomas

Although *IDH* mutations often occur in gliomas, not all gliomas carry *IDH* mutations. The gain-of-function mutation shifts IDH activity. Its mutant IDH mediates the conversion of αKG into 2HG.

### Isocitrate dehydrogenase (IDH)

One of the most exciting discoveries of the past years in the field of cancer metabolism was the one of IDH1/2 mutations in gliomas and acute myeloid leukemia (Dang et al. 2010; Reitman and Yan 2010). Only few mutations in genes that encode for metabolic enzymes are linked to tumor genesis. Examples for homozygous loss-of-function mutations in metabolic tumor suppressors are fumarate hydratase or one of the five subunits comprising the succinate dehydrogenase complex (King et al. 2006). In contrast to these examples, heterozygous IDH mutations lead to a gain-of-function. Therefore, *IDH* is not a tumor suppressor gene.

Due to the great number of recent reviews on this topic (Guo et al. 2011; van Lith et al. 2014; Molenaar et al. 2014; Bogdanovic 2015; Borodovsky et al. 2015; Parker and Metallo 2015), this report will be limited to a brief summary of main observations on *IDH* mutations and the consequence for cell metabolism in regard to Glu and αKG.

### 2-Hydroxyglutarate is an oncometabolite

IDH enzymes catalyze the NADP^+^/NAD^+^-dependent conversion of isocitrate to 2HG (also known as 2-oxoglutarate; Zhao et al. 2009). The cytoplasmic version of the enzyme, IDH1, acts in the cytoplasm; IDH2 and IDH3 act in mitochondria, mainly in the TCA cycle. 70–90 % of grades II and III glioma and secondary GBMs carry mutated *IDH1* or *IDH2* genes (Parsons et al. 2008; Yan et al. 2009). Mutations in the *IDH3* gene have not been reported in connection with tumors; therefore, in this article the term *IDH* mutation refers only to *IDH1* and *IDH2*. The most common amino acid substitution in glioma is the replacement of arginine with histidine in the *IDH1* gene (IDH1R132H; Parsons et al. 2008; Yan et al. 2009; Borger and Zhu 2012; Hirata et al. 2015, Table 1). In the case of IDH2, the substrate-binding arginine (Arg) residues Arg 140 and Arg 172 are mutated. In both cases the mutation results in a gain-of-function of the enzyme, which leads to an increased conversion of αKG to 2HG and subsequently to an accumulation of 2HG in the cell (Dang et al. 2009; Gross et al. 2010; Ward et al. 2010). 2HG itself inhibits Ten-Eleven-Translocation (TET) family and Jumonji-C-domain-containing histone demethylases (JHDMs; Chowdhury et al. 2011; Xu et al. 2011; Fig. 3).

**Table 1:** Differences between wild-type and mutated IDH

	Wild-type *IDH*	Mutant *IDH*
Heterozygous point mutations in the catalytic site	None	Arg to His in position 132 (*IDH1*, R132H) Arg in position 172 and 140 (*IDH2*)
Substrate affinity	Same affinity for isocitrate and α-ketoglutarate	Lower affinity for isocitrate, higher affinity for α-ketoglutarate
IDH-mediated reaction	Isocitrate <−> *α*-ketoglutarate	α-Ketoglutarate—>2-hydroxyglutarate
2-hydroxyglutarate levels	Low	High

These enzymes are directly involved in demethylating processes in the genome. In that way high 2HG concentrations in the cell lead to global DNA hypermethylation and altered gene expression (Figueroa et al. 2010; Sasaki et al. 2012; Turcan et al. 2012). Unsurprisingly there is a tight correlation between *IDH1* mutations and a hypermethylated phenotype named glioma-CpG island methylator phenotype (G-CIMP; Noushmehr et al. 2010). Since these alterations provide the basis for cancer, 2HG is described as ‘oncometabolite’. The exciting fact about *IDH* mutations is that it seems likely that this metabolic alteration is more of a major contributor to cancer initiation and progression, than a mere side effect. This makes it a potential target for therapy. Inhibitors of mutant IDH1 have been effective in lowering 2HG levels in vivo (Popovici-Muller et al. 2012) and reducing growth of glioma cells in vitro (Rohle et al. 2013). However, there is more to IDH-mutant cancer cells than just a shift in levels of two metabolites.

### αKG and Glu in IDH-mutant cancer cells

αKG levels should be low in *IDH*-mutant cancer cells compared to *IDH* wt cells, due to increased conversion of αKG to 2HG. In fact they are relatively high, because mitochondrial biosynthesis makes up for missing cytoplasmic αKG (Van Lith et al. 2014). That in turn influences the mitochondrial TCA cycle considerably (Table 2).

**Table 2:** Versatile effects of *IDH* mutations on cell metabolism

Alterations through *IDH* mutation	Affected pathway	Negative effect on
αKG to 2HG-conversion	αKG–2HG	NADPH production
Increased 2HG levels	αKG-2HG	DNA methylation ATP synthesis
Increased need for cytoplasmic αKG	Glu-αKG	Glu export Cys import
	Mitochondrial malate–αKG antiporter	αKG levels in mitochondria
Reduced αKG availability	Glutaminolysis	ATP production
Distorted conversion of αKG to isocitrate	Reductive carboxylation	Lipid synthesis

### Mutant IDH results in deployed lipid synthesis

Increased synthesis of αKG through mitochondria means decreased levels of αKG substrates like citrate. Citrate, however, is crucial for acetly-CoA synthesis and therefore directly involved in the build-up of fatty acids. It is conceivable that the mutation of IDH leads to an altered ratio of fatty acid products like sphingolipids, or phospholipids. Although no data have been published on this matter, it has been claimed that *IDH1* mutations indirectly alter the levels of sphingomyelin in mouse brains (Bogdanovic 2015).

As described earlier, IDH does not only assist the conversion of isocitrate to αKG, but also the reverse reaction during reductive carboxylation. In *IDH*-mutant tumors one would expect reductive carboxylation to be idle, due to the partial loss of IDH activity. Indeed, in hypoxic conditions heterozygous *IDH1*-mutant cells were impeded in their reductive carboxylation ability and increased their oxidative TCA metabolism (Table 3). *IDH2* mutant cells, however, continued to perform reductive carboxylation (Grassian et al. 2014). It was shown that even an abundant amount of substrate for reductive carboxylation in *IDH1* mutant cells did not result in higher citrate levels, which suggest that the process itself is disrupted. *IDH1* mutant glioma cells might be sensitive to mitochondrial stress, because the *IDH1* mutation silences the cytoplasmic, Gln-dependent pathway for metabolites and fatty acids (Table 3).

**Table 3:** Lipids synthesis in cells with wild-type *IDH* and mutated *IDH* under different growth conditions

	Wild-type *IDH*	Mutant *IDH*
IDH-mediated reaction	Isocitrate <−> α-ketoglutarate	α-ketoglutarate—>2-hydroxyglutarate
Lipid synthesis under		
Normal conditions	Good	Good
Main substrate	Glucose	Glucose
Pathway	TCA cycle	TCA cycle
Hypoxic conditions	Good	Compromised
Main substrate	Glutamine	Glutamine
Pathway	IDH-mediated reversed flux	Disturbed reversed flux
Inhibited TCA metabolism	Good	Compromised
Main substrate	Glutamine	Glutamine
Pathway	IDH-mediated reversed flux	Disturbed reversed flux

### IDH-mutant cells are susceptible to ROS-induced damage

In cases of disrupted metabolic pathways, like an impeded TCA cycle, it is crucial for the cell to obtain a TCA cycle-independent source for αKG. Glu can easily be converted into αKG in the cytoplasm. Contrary to IDH wt glioma cells, IDH-mutant glioma cells do not release Glu on a large scale, because it is needed among others as substrate for the TCA cycle. Since IDH-mutant gliomas are “glutamate suckers” (Van Lith et al. 2014), they cannot import much Cys through the system *x*_c_^−^. Therefore, they might have a lack of GSH. Together with low levels of NADPH this can result in a hampered resistance to ROS. Since Glu levels are strongly affiliated with Gln levels, IDH-mutant glioma cells also experience a high demand for Gln.

**Fig. 4:**
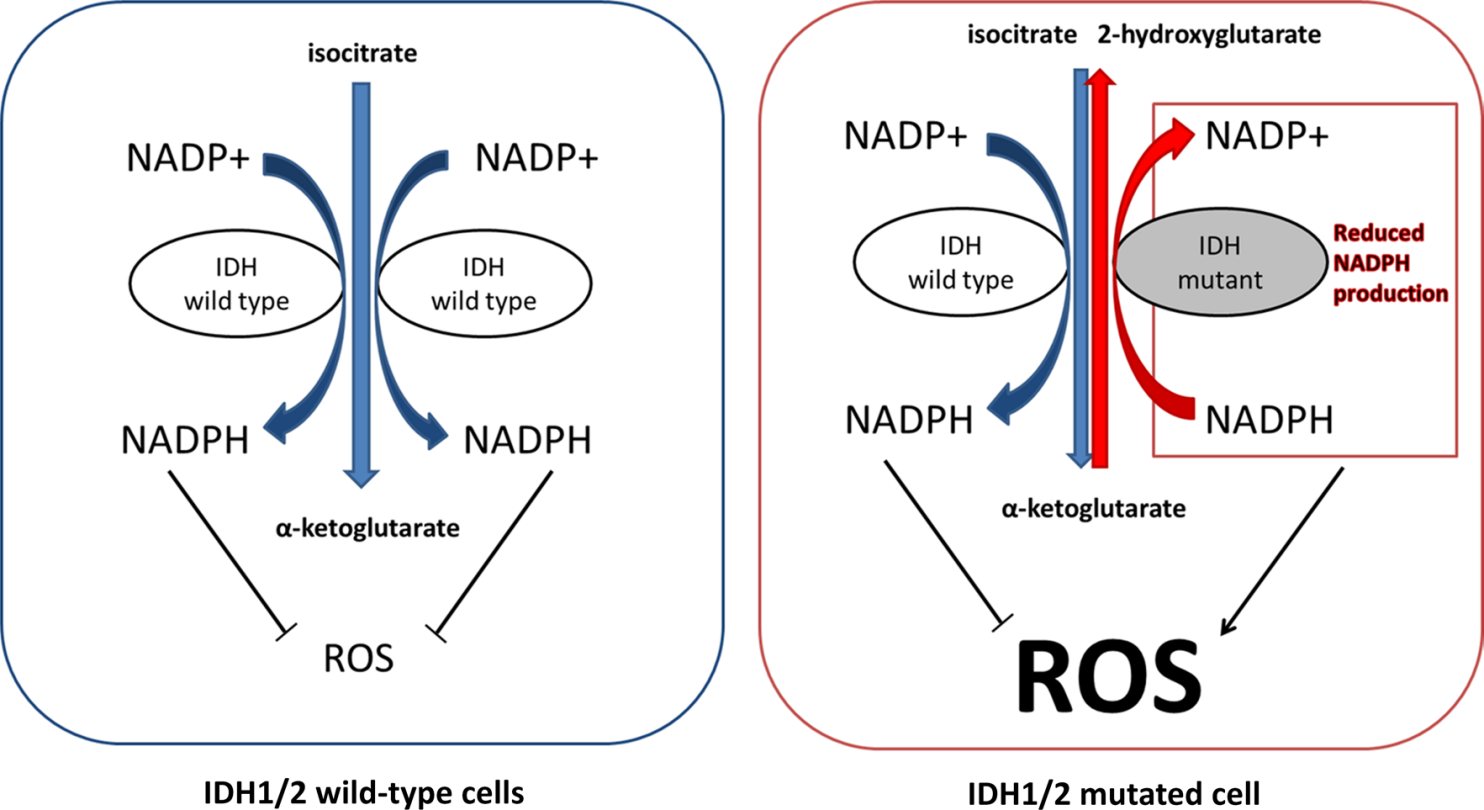
In *IDH1/2* wild-type cells, continuous NADPH production ensures low ROS levels (*left*). Mutant IDH1/2 activity consumes NADPH and lowers NADPH production. That results in increased ROS levels (*right*). *ROS* reactive oxygen species

The IDH1-mediated conversion of αKG to isocitrate goes along with the conversion of NADPH to NADP^+^. The reversed reaction creates NADPH. Mutant IDH1, however, does not mediate reactions with isocitrate anymore. Instead it mediates the unidirectional conversion of αKG into 2HG with NADP^+^ as abundant side product. Subsequently cytoplasmic production of NADPH or NADH, respectively, is decreased and available NADPH or NADH is consumed (Fig. 4). NADPH is the major metabolite to protect the cell from reactive oxygen species (ROS), because it is necessary to turn oxidized glutathione (GSSG) into reduced glutathione (GSH). GSH in turn directly neutralizes free radicals and ROS.

As stated above, it is assumed that increased mitochondrial IDH2 activity makes up for cytoplasmic lack of αKG, which would increase NADPH availability. To what extent mitochondrial NADPH is transported to the cytoplasm remains to be elucidated. Whether the cancers with mutant *IDH* are more susceptible to ROS-induced stress (e.g., through radiation or temozolomide) is certainly dependent on the effects of the altered metabolism on the pentose phosphate pathway (PPP). The PPP is the major source of NADPH in the cytoplasm (Eggleston and Krebs 1974), and has been linked to cancer metabolism (Tsouko et al. 2014).

## 2HG directly interferes with ATP synthase

Besides the tumor-initiating effects, both enantiomers of 2HG have another substantial effect on cancer cell metabolism. 2HG and αKG are capable of binding directly to ATP synthase. In this way the enzyme is inhibited and cannot exert ATP synthesis anymore. That has negative effects on mitochondrial respiration and mTOR signaling in *ID-* mutant cancers (Fu et al. 2015).

## Conclusion

In this review we highlight that αKG and Glu are keystones in several crucial metabolic pathways. Next to glucose, Gln and αKG are important energy sources for the cancer cell. Especially in hypoxic conditions glutaminolysis and reductive carboxylation are needed to sustain cancer cell growth and proliferation. Glu and αKG are important links in the sequence of biochemical reactions. Furthermore, released Glu can have an impact on cells in the tumor vicinity, since high Glu levels lead to astrocytic swelling and apoptosis, which is believed to ease tumor expansion. Blockage of the efflux transporter System *x*_c_^−^ abrogates the negative effect of glioma cells on their microenvironment. However, it does not hamper the glioma cells’ viability. Similarly, deprivation of amino acids has negative effects on cancer cells, but this is not sufficient to cause apoptosis. For example, Asn alone can block the apoptotic pathway on Glu-deprived glioma cells. Interestingly, when amino acids like Cys or Met are lacking in glioma cells, ROS levels increase. When it would be possible to push ROS levels over a critical limit, glioma proliferation would be inhibited. To achieve this, a combined approach of radiation, temozolomide and deprivation of amino acids and nutrients would be optimal. However, it is questionable if this is applicable in a clinical setting, without severely damaging healthy areas of the brain, regarding the neurotoxic side effects of anti-cancer drugs such as oxaliplatin, bortezomib, or epothilone-B (Ceresa et al. 2014).

Recently a meta-study of 55 observational studies has shown that glioma patients with *IDH*-mutant tumors have a higher overall survival rate compared to *IDH*-wt tumors. Furthermore their progression-free survival is significantly increased (Xia et al. 2015). This report is in line with reports of higher degrees of cell death in *IDH*-mutant gliomas. Regarding the reports about metabolic changes through *IDH* mutations, it seems natural that tumors with *IDH* mutations perform worse than *IDH*-wt tumors. Here we summarize the ways in which *IDH* mutations impede cellular metabolism:

1. Mutant *IDH1* cannot perform reductive carboxylation to allow macromolecule synthesis in hypoxia.
2. Mutant *IDH* enzymes enhance the NADPH-dependent conversion of αKG to 2HG; leaving the cell with low NADPH levels and therefore more susceptible to stress through free radicals and ROS.
3. 2HG directly inhibits ATP synthase by binding to it and leads to unfavorable effects under glucose restriction.

It became apparent that especially hypoxic conditions are highly unfavorable for *IDH*-mutant cells. *IDH*-mutant cells might not be able to survive or at least proliferate in hypoxic conditions (Fig. 3). That would explain why IDH mutations are not common in solid tumors and only have been documented in diffuse forms of cancer like glioma or leukemia. In diffuse cancer types cells do not stay in close vicinity and therefore have fewer problems with oxygen supply.

*IDH* mutations play a large role in tumor onset. Later on, however, the malfunction of the IDH enzyme seems to challenge the cell more than it aids it. The metabolic alterations discussed in this article give a partial explanation at a molecular level for the effects of *IDH* mutations on overall survival that were reported in clinical studies. With regards to therapy approaches, it might be effective to enhance the problems the cell is confronted with by the mutant IDH enzyme.

## Abbreviations

2HG: *2*-Hydroxyglutarate (also known as 2-oxoglutarate)
ALL: Acute lymphoblastic leukemia
Arg: Arginine
AS: Asparagine synthetase
Asn: Asparagine
ASNase: Asparaginase
BCAT1: Branched-chain amino acid transaminase 1
Cys: Cysteine
EGFR: Epidermal growth factor receptor
G-CIMP: Glioma-CpG island methylator phenotype
GBM: Glioblastoma multiforme
GDH: Glutamate dehydrogenase
GLA: Glutaminase
Gln: Glutamine
GLS: *Gls* gene
Glu: Glutamate
GSH: (reduced) Glutathione
GSSG: (oxidized) glutathione
IDH1: Isocitrate dehydrogenase 1
IDH2: Isocitrate dehydrogenase 2
JHDMs: Jumonji-C-domain-containing histone demethylases
Met: Methionine
MRS: Magnetic resonance spectroscopy
mTOR: Mechanistic target of rapamycin (or mammalian target of rapamycin)
mTORC1: mTOR complex 1
mTORC2: mTOR complex 2
PDH: Pyruvate dehydrogenase
PPP: Pentose phosphate pathway
ROS: Reactive oxygen species
siRNA: Small interfering RNA
TCA: Tricarboxylic acid
TET: Ten-eleven-translocation
wt: Wild-type
αKG: α-Ketoglutarate
